# Progesterone levels on hCG trigger day and neonatal outcomes in fresh embryo transfers: a retrospective threshold analysis of GnRH antagonist versus agonist long protocols

**DOI:** 10.3389/fendo.2026.1982427

**Published:** 2026-10-07

**Authors:** Jing Wu, Hengde Zhang, Jianlei Huang, Li Hai, Xinxin Liang, Dan Liu, Jing Zhou, Xuan Li, Yuping He, Xiaohong Wang

**Affiliations:** Reproductive Medicine Center, Department of Obstetrics and Gynecology, Tangdu Hospital, Fourth Military Medical University, Xi’an, China

**Keywords:** progesterone level, fresh embryo transfer, GnRH antagonist protocol, neonatal outcomes, preterm birth, low birthweight

## Abstract

**Background:**

Elevated progesterone levels on the day of human chorionic gonadotropin (hCG) trigger are known to adversely affect pregnancy outcomes in fresh embryo transfer (fET) cycles, but their impact on neonatal outcomes remains poorly understood. This study aimed to investigate the association between progesterone levels on hCG trigger day and neonatal outcomes, and to explore the progesterone threshold, so as to provide reference for clinical decision-making.

**Methods:**

This retrospective cohort study included 2, 201 patients who achieved singleton live births following fET between April 1, 2021 and December 31, 2024. All patients were ≤ 42 years of age and underwent either a GnRH antagonist protocol or an agonist long protocol. A multivariable regression analysis was performed to detect the relationship between progesterone level and neonatal outcomes. Smooth curve fitting and threshold effect analysis were used to evaluate the cutoff value of progesterone level.

**Results:**

In the overall population, higher progesterone levels were significantly associated with increased risks of preterm delivery (PTD) (adjusted OR: 1.89, 95% CI: 1.03 to 3.47, P = 0.0399) and low birthweight (LBW) (adjusted OR: 2.67, 95% CI: 1.14 to 6.24, P = 0.0231). These associations were observed only in the GnRH antagonist protocol, particularly with progesterone ≥ 1.0 ng/mL significantly elevating the risks of PTD (adjusted OR: 4.54, 95% CI: 2.16 to 9.56, P = 0.0021) and LBW (adjusted OR: 4.16, 95% CI: 1.15 to 15.07, P = 0.0378). Threshold analysis further identified progesterone > 0.90 ng/mL as the inflection point for PTD (adjusted OR: 6.60, 95% CI: 2.52 to 14.56, P = 0.0131) and progesterone > 0.98 ng/mL for LBW (adjusted OR: 7.50, 95% CI: 3.71 to 15.87, P = 0.0038) in antagonist cycles. No such associations were found in the GnRH agonist long protocol.

**Conclusions:**

Our findings suggest that in GnRH antagonist cycles, a serum progesterone level exceeding 0.90 ng/mL on hCG trigger day may serve as an independent predictor of an increased risk of PTD in singletons born after fET. These results highlight the need for enhanced perinatal surveillance in this population, and call for further validation in larger prospective multicenter studies.

## Introduction

Since the first live birth following *in vitro* fertilization and embryo transfer (IVF-ET) in 1978, IVF has become a cornerstone in the treatment of infertility, offering couples the opportunity to conceive when natural conception fails. Upon the introduction of controlled ovarian stimulation (COS), continuous efforts have been made to optimize stimulation protocols and better understand their physiological basis, aiming to improve both the efficacy and safety of IVF. In this context, the significance of progesterone levels on the day of human chorionic gonadotropin (hCG) administration in gonadotropin releasing hormone (GnRH) analogue cycles, as a vital factor in assessing oocyte maturity and promoting the transformation of endometrium to secretory phase, has garnered significant attention for nearly three decades.

Extensive research has demonstrated that elevated progesterone levels on the hCG trigger day would have a negative impact on IVF pregnancy outcomes (1–8). Based on a meta-analysis of over 60, 000 cycles, Venetis et al. reported that when the threshold was defined as ≥ 0.8 ng/ml, an elevated progesterone level on hCG trigger day significantly reduced the probability of pregnancy in fresh IVF cycles (9). A recent review also suggested that, despite varying thresholds in different studies, the available data generally supported that elevated progesterone levels on the day of hCG administration may adversely affect the success of IVF pregnancies (4). The underlying mechanisms of the detrimental effect have been confirmed to potentially exert their influence through the action of elevated progesterone levels on the endometrial receptivity, leading to asynchrony at the embryo-endometrium crosstalk, therefore impairing the implantation process (8, 10–16). The other explanation would be impaired oocyte quality and reduced the rate of high-quality embryos (17–21).

Given the close associations of embryo-endometrial asynchrony and compromised oocyte and embryo quality with neonatal outcomes, such as low birth weight (LBW) or preterm delivery (PTD), we hypothesized that elevated progesterone levels on hCG trigger day may play an important role in determining neonatal outcomes. However, research on this issue remains extremely limited. To date, only Ibrahim et al. have reported that an elevated progesterone level on the hCG trigger day during fresh embryo transfer (fET) cycles, particularly when exceeding 2.0 ng/mL, may lead to a decrease in birthweight, yet their study focused on two main outcome measures: birthweight and Z-score (22). This limited evidence leaves a significant gap in the comprehensive understanding of neonatal safety as affected by elevated progesterone levels. To address this gap, the present study aimed to investigate the association between progesterone levels on the hCG trigger day and neonatal outcomes following fET. To minimize the confounding effect of COS protocols, a major confounder in this study, we further performed multivariate regression analysis stratified by COS protocols to validate this relationship and enhance the robustness of our findings. Furthermore, if elevated progesterone levels on the hCG trigger day are indeed harmful to neonatal outcomes, the progesterone threshold above which the risk of adverse neonatal outcomes becomes significantly elevated has not yet been reported. This study is the first to evaluate the exploratory threshold of serum progesterone levels on hCG trigger day affecting neonatal outcomes using adjusted smooth curve fitting and threshold effect analysis, ultimately aiming to provide guidance for clinical decision-making.

## Methods

### Study population

This retrospective cohort study was conducted at the Assisted Reproduction Center of Tangdu Hospital, Fourth Military Medical University, China, and included data from April 1, 2021, to December 31, 2024. We analyzed cycles that met the following inclusion criteria (1): women aged ≤ 42 years at oocyte retrieval (2); autologous IVF or intracytoplasmic sperm injection (ICSI) (3); COS using either a long gonadotropin-releasing hormone agonist (GnRH-a) protocol or a GnRH antagonist protocol (4); within the first three oocyte retrieval cycles; and (5) live singleton birth following fET. We excluded patients with polycystic ovary syndrome (PCOS) diagnosed according to the Rotterdam criteria, as well as those with prepregnancy hyperglycemia, hypertension, or thyroid dysfunction, to minimize confounding effects on neonatal outcomes. In addition, cycles involving multiple gestations, vanishing twin syndrome, uterine malformations, cervical incompetence, a history of intrauterine or cervical surgery, or missing data on progesterone levels were also excluded. The patient selection process is shown in **Figure 1**. Ultimately, 2, 201 cycles were included in the final analysis.

**Figure 1:**
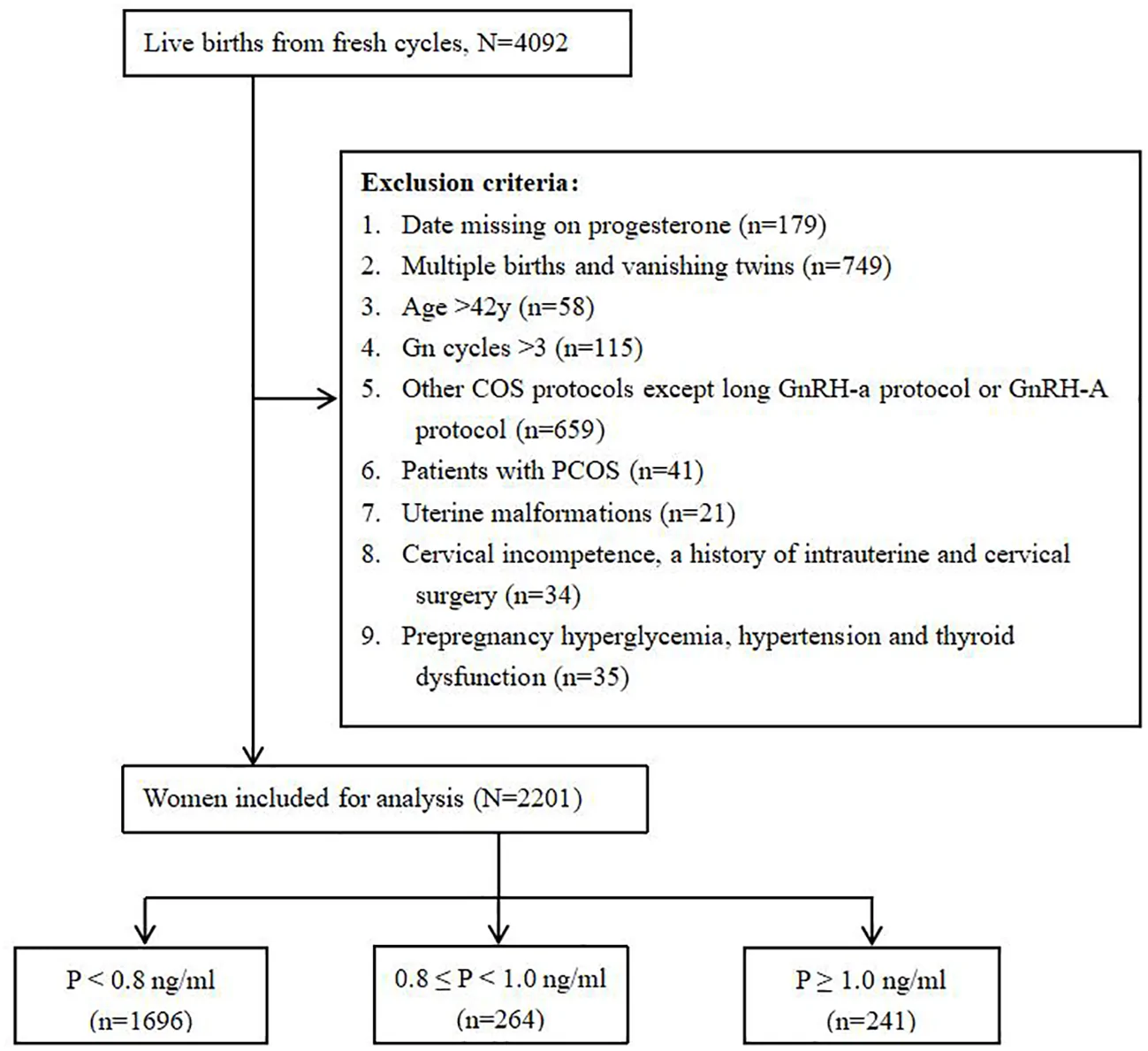
Flowchart of patient inclusion. Gn, gonadotrophin; COS, controlled ovarian stimulation; GnRH-a, GnRH agonist; GnRH-A, GnRH antagonist; P, progesterone; PCOS, polycystic ovarian syndrome.

### Treatment procedures

Ovarian stimulation protocols were uniformly conducted using either the long GnRH-a protocol or the antagonist protocol. The initiating gonadotropin (Gn) dosage and the selection of COS protocol were determined based on patient characteristics, hormone levels and follicular diameter on the day of Gn initiation, as well as the physician’s experience. Recombinant or urinary-derived exogenous Gn was administered to all participants. The daily dosage of Gn was adjusted according to follicular development and hormonal changes, as monitored by serial transvaginal ultrasound examinations and serum hormone level assessments, including estradiol (E2), progesterone, and luteinizing hormone (LH), until the day of hCG administration to induce ovulation. Blood samples were collected from patients between 7 a.m. and 9 a.m. during the COS procedure. The levels of serum hormones were assayed using the Immulite 2000 Immunoassay System manufactured by Siemens Healthcare Global, featuring a sensitivity of 0.1 ng/mL for progesterone and 15 pg/mL for E2. This assay was utilized throughout the entire duration of the study. In all patients, ovulation was triggered using 250 μg of recombinant human chorionic gonadotropin (rhCG) or 5000–10000 IU of urinary hCG once transvaginal ultrasound revealed at least three follicles with a diameter of ≥17 mm or at least two follicles with a diameter of ≥18 mm. Oocyte retrieval was conducted under transvaginal ultrasound guidance 35–37 hours after hCG administration. Fertilization assessment of the oocytes was performed approximately 12 to 17 hours following insemination or sperm injection. Cleavage stage embryo transfers occurred at the third day, whereas blastocyst-stage transfers were arranged for the fifth day subsequent to oocyte retrieval (23). In our center, fET is routinely canceled when progesterone levels on the hCG trigger day exceed 1.5 ng/ml or when a GnRH agonist trigger is used alone. Throughout the duration of the study, no significant alterations were observed in clinical and laboratory conditions, the culture media utilized for embryo growth, or the fET techniques.

### Outcome measures and definitions

Information on patients’ baseline characteristics and parameters of the COS cycle was gathered, including the couples’ ages, maternal body mass index (BMI), antimüllerian hormone (AMH) levels, type of infertility, infertility duration and cause, prior Gn cycles, COS protocols, total Gn dose, length of stimulation, E2 and progesterone levels on the day of hCG trigger, number of oocytes retrieved, developmental stages and numbers of embryos transferred, and thickness and type of endometrium. Data regarding the height, weight, sex, gestational age (GA), and delivery method of newborns was documented for every live birth. GA was calculated starting from the day of embryo transfer, which was designated as Day 17 for transfers involving cleavage-stage embryos and Day 19 for those involving blastocyst-stage embryos (24).

Preterm delivery (PTD) and very PTD were defined as GA of 32–36 weeks and less than 32 weeks, respectively. Low birth weight (LBW), very LBW, and macrosomia were identified as birthweight of less than 2, 500 g, less than 1, 500 g, and 4, 000 g or more, respectively. Small for gestational age (SGA) and very SGA were defined as birthweight below the 10th and 3rd percentiles. Large for gestational age (LGA) and very LGA were defined as birthweight above the 90th and 97th percentiles, respectively. Additionally, the Z-score was introduced to adjust birthweight for GA and neonatal sex, calculated using the equation: Z-score = (x - μ)/σ, in which x is the newborns’ weight, and μ and σ are the average birthweight and the standard deviation of infants of the same sex and the same GA, respectively. The birthweight percentiles and Z-score calculations were referenced from Chinese singleton newborns’ data, stratified by GA and neonatal sex (25).

### Statistical analysis

Continuous variables are presented as means ± standard deviation (SD) or medians with interquartile ranges (IQR), as appropriate. Categorical variables are expressed as numbers (percentages). For comparisons between groups, continuous variables were analyzed using one-way analysis of variance (ANOVA), and categorical variables were compared using the chi-square test or Fisher’s exact test, as appropriate.

Multiple linear regression models were constructed to examine the associations of serum progesterone level (ng/mL) with birthweight (g), GA (weeks), and birthweight Z-score. Multiple logistic regression analyses were performed to evaluate the associations with categorical outcomes, including LBW, very LBW, fetal macrosomia, preterm, very preterm, SGA, very SGA, LGA, and very LGA, with adjustment for potential confounding variables. For outcomes that demonstrated statistically significant associations, protocol-stratified analyses were further conducted to minimize confounding by COS regimens. Additionally, smooth curve fitting and threshold effect analyses were performed to explore the threshold of serum progesterone level on the day of hCG trigger above which the risks of LBW and PTD were significantly increased.

Potential confounding variables were selected based on their clinical relevance, their associations with the outcomes of interest, or a change in the effect estimate of >10% upon inclusion in the model. In the regression models for birthweight-related outcomes (including fetal macrosomia, LBW, very LBW, SGA, very SGA, LGA, and very LGA), adjustments were made for maternal BMI, AMH, duration of infertility, cause of infertility, COS protocol, total Gn dose, serum E2 level on the day of hCG trigger, endometrial thickness, stage of embryos transferred, GA, and newborn sex. For the models examining GA, PTD, and very PTD, we adjusted for maternal BMI, AMH, duration of infertility, cause of infertility, COS protocol, endometrial thickness, stage of transferred embryos, and newborn sex.

All statistical analyses were performed using EmpowerStats (X&Y Solutions, Inc., Boston, MA, USA) and R software version 3.6.1 (The R Foundation for Statistical Computing, Vienna, Austria). A P value of <0.05 was considered to be statistically significant.

## Results

A total of 2, 201 fresh IVF cycles were included in this study and were stratified into three groups according to the previously published cutoff values for progesterone levels on the day of hCG trigger (2, 8, 9). Among them, 1, 696, 264, and 241 live-born singletons were categorized as Group 1 (progesterone < 0.8 ng/mL), Group 2 (0.8 ≤ progesterone < 1.0 ng/mL), and Group 3 (progesterone ≥ 1.0 ng/mL), respectively.

The patients baseline characteristics, COS cycle parameters and neonatal outcomes are shown in **Table 1**. Compared with Group 1, women in the higher progesterone groups had lower maternal BMI and lower AMH concentration. With respect to COS cycle parameters, the higher groups of progesterone level were administered a greater total dosage of Gn, underwent a longer duration of stimulation, displayed higher E2 levels on the hCG trigger day, more cleavage-stage embryos transferred and thinner endometrium. In terms of neonatal outcomes, with increasing progesterone level, birthweight (g) and GA (weeks) gradually decreased, and the proportion of LBW, PTD and caesarean section increased. The differences in all of the above mentioned indices were significant (P<0.05).

**Table 1:** Patient baseline characteristics, COS cycle parameters and neonatal outcomes by different groups of progesterone level on hCG trigger day (n=2201).

Characteristics	Progesterone < 0.8ng/ml	0.8 ≤ Progesterone < 1.0ng/ml	Progesterone ≥ 1.0ng/ml	*P*-value
	(n=1696)	(n=264)	(n=241)	
Baseline characteristics				
**Maternal age (y)**	31.49 ± 3.89	31.91 ± 4.04	32.00 ± 3.85	0.066
**Paternal age (y)**	32.86 ± 4.52	33.22 ± 4.66	33.32 ± 4.68	0.219
**Maternal BMI (kg/m^2^)**	22.63 ± 3.16	22.17 ± 2.89	22.23 ± 3.16	**0.029**
**AMH (ng/ml)**	2.99 ± 2.08	2.43 ± 1.85	2.44 ± 1.64	**<0.001**
**Type of infertility**				0.200
Primary	815 (48.05%)	139 (52.65%)	108 (44.81%)	
Secondary	881 (51.95%)	125 (47.35%)	133 (55.19%)	
**Infertility duration (y)**	3.53 ± 2.78	3.64 ± 2.87	3.47 ± 2.95	0.766
**Infertility cause**				0.493
Female	974 (60.84%)	153 (60.71%)	146 (63.20%)	
Male	285 (17.80%)	37 (14.68%)	32 (13.85%)	
Mixed	261 (16.30%)	44 (17.46%)	42 (18.18%)	
Unexplained	81 (5.06%)	18 (7.14%)	11 (4.76%)	
**Prior gonadotropin cycle**	1.16 ± 0.43	1.15 ± 0.38	1.26 ± 0.49	0.080
COS cycle parameters				
**COS protocols**				0.062
GnRH-a long protocol	1111 (65.51%)	176 (66.67%)	131 (54.36%)	
Antagonist protocol	585 (34.49%)	88 (33.33%)	110 (45.64%)	
**Dosage of gonadotropins (IU)**	1997.97 ± 975.48	2434.28 ± 1154.33	2531.33 ± 1100.71	**<0.001**
**Stimulation duration (days)**	11.54 ± 1.97	12.02 ± 2.02	12.01 ± 2.02	**<0.001**
**LH level on hCG day (mIU/ml)**	2.99 ± 2.95	2.72 ± 1.68	2.90 ± 2.45	0.744
**E2 level on hCG day (pg/ml)**	2462.76 ± 1132.87	2832.15 ± 1061.55	2723.38 ± 1038.29	**<0.001**
**Progesterone level on hCG day** **(ng/ml)**	0.49 ± 0.17	0.89 ± 0.05	1.22 ± 0.23	**<0.001**
**Number of oocytes retrieved**	9.63 ± 3.62	9.87 ± 3.35	10.05 ± 3.65	0.185
**Fertilization method**				0.190
IVF	1118 (65.92%)	163 (61.74%)	149 (61.83%)	
ICSI	505 (29.78%)	83 (31.44%)	76 (31.54%)	
IVF+ICSI	73 (4.30%)	18 (6.82%)	16 (6.64%)	
**Number of embryos transferred**				0.098
1	906 (53.42%)	132 (50.00%)	98 (40.66%)	
2	790 (46.58%)	132 (50.00%)	143 (59.34%)	
**Endometrial thickness (mm)**	10.48 ± 1.71	10.52 ± 1.71	10.09 ± 1.52	**0.003**
**Endometrial type**				0.095
A	38 (2.27%)	8 (3.04%)	0 (0.00%)	
A-B	68 (4.06%)	11 (4.18%)	6 (2.51%)	
B	598 (35.70%)	82 (31.18%)	73 (30.54%)	
B-C	830 (49.55%)	139 (52.85%)	135 (56.49%)	
C	141 (8.42%)	23 (8.75%)	25 (10.46%)	
Neonatal outcomes				
**Newborn sex**				0.729
Male	918 (54.13%)	136 (51.52%)	129 (53.53%)	
Female	778 (45.87%)	128 (48.48%)	112 (46.47%)	
**Newborn height (cm)**	50.07 ± 2.61	49.99 ± 2.57	49.71 ± 2.60	0.133
**GA (weeks)**	38.99 ± 1.69	38.85 ± 1.74	38.71 ± 1.90	**0.045**
**Birthweight (g)**	3292.73 ± 503.30	3226.52 ± 519.66	3216.96 ± 535.74	**0.023**
**Z-score**	0.23 ± 1.08	0.11 ± 1.00	0.16 ± 1.03	0.164
**Very low birthweight (<1500g)**	11 (0.65%)	4 (1.52%)	2 (0.83%)	0.325
**Low birthweight (<2500g)**	73 (4.30%)	15 (5.68%)	20 (8.30%)	**0.003**
**Fetal macrosomia (≥4000g)**	123 (7.25%)	17 (6.44%)	11 (4.56%)	0.291
**Very preterm (<32w)**	17 (1.00%)	3 (1.14%)	3 (1.24%)	0.930
**Preterm (32-36w)**	44 (2.59%)	13 (4.92%)	15 (6.22%)	**0.022**
**Small for gestational age**	101 (5.96%)	17 (6.44%)	21 (8.71%)	0.256
**Very small for gestational age**	37 (2.18%)	8 (3.03%)	6 (2.49%)	0.683
**Large for gestational age**	132 (7.78%)	19 (7.20%)	11 (4.56%)	0.267
**Very large for gestational age**	69 (4.07%)	7 (2.65%)	6 (2.49%)	0.389
**Mode of delivery**				**0.005**
Vaginal	611 (36.28%)	82 (31.30%)	63 (26.36%)	
Caesarean section	1073 (63.72%)	180 (68.70%)	176 (73.64%)	

COS, controlled ovarian stimulation; GnRH-a, GnRH agonist; BMI, body mass index; AMH, antimüllerian hormone; E2, estradiol; LH, luteinizing hormone. Bold values indicate: P < 0.05, which is statistically significant.

The univariate linear analyses outlined in **Table 2** indicated that five factors had a significant impact on birthweight (g): maternal BMI, E2 level on the day of hCG administration, endometrial thickness, newborn sex, and GA. Additionally, three factors were found to significantly influence GA (w): maternal BMI, endometrial thickness and newborn sex.

**Table 2:** Univariate analysis of impact factors on birthweight and gestational age (n=2201).

Exposure	Change in birthweight (g)		Change in gestational age (w)	
	β(95%CI)	*P* value	β(95%CI)	*P* value
**Maternal age (y)**	2.00 (-5.16, 9.16)	0.5841	-0.02 (-0.04, 0.01)	0.1584
**Paternal age (y)**	2.15 (-3.78, 8.08)	0.4774	-0.02 (-0.04, 0.00)	0.1028
**Maternal BMI (kg/m^2^**)	16.78 (7.46, 26.10)	**0.0004**	-0.03 (-0.07, -0.00)	**0.0463**
**AMH (ng/ml)**	-0.38 (-13.51, 12.75)	0.9550	0.02 (-0.02, 0.07)	0.3657
Type of infertility				
Primary	Ref		Ref	
Secondary	28.75 (-27.57, 85.06)	0.3172	-0.14 (-0.33, 0.06)	0.1782
**Infertility duration (y)**	-8.95 (-18.55, 0.66)	0.0681	-0.03 (-0.06, 0.01)	0.0973
Infertility cause				
Female	Ref		Ref	
Male	-2.40 (-72.23, 67.43)	0.9463	0.11 (-0.13, 0.36)	0.3665
Mixed	-22.72 (-106.63, 61.19)	0.5957	-0.08 (-0.37, 0.22)	0.6075
Unexplained	-57.06 (-173.58, 59.46)	0.3373	-0.08 (-0.49, 0.33)	0.6946
**Prior gonadotropin cycle**	-5.12 (-67.06, 56.82)	0.8714	-0.11 (-0.32, 0.11)	0.3191
COS protocols				
GnRH-a long protocol	Ref		Ref	
Antagonist protocol	-32.75 (-91.77, 26.27)	0.2769	-0.19 (-0.40, 0.02)	0.0730
**Dosage of gonadotropins (IU)**	0.02 (-0.01, 0.04)	0.2197	-0.00 (-0.00, 0.00)	0.4692
**Stimulation duration (days)**	2.47 (-11.66, 16.60)	0.7316	0.01 (-0.04, 0.06)	0.7830
**E2 level on hCG day (pg/ml)**	-0.04 (-0.06, -0.01)	**0.0017**	0.00 (-0.00, 0.00)	0.8048
**LH level on hCG day (mIU/mL)**	0.55 (-16.72, 17.82)	0.9502	0.00 (-0.06, 0.06)	0.9157
**Progesterone level on hCG day (ng/ml)**	-173.57 (-274.13, -73.02)	**0.0007**	-0.33 (-0.68, 0.03)	0.0715
**Number of oocytes retrieved**	0.54 (-7.18, 8.25)	0.8912	0.02 (-0.01, 0.05)	0.1482
Fertilization method				
IVF	Ref		Ref	
ICSI	-21.39 (-83.95, 41.17)	0.5029	-0.02 (-0.24, 0.20)	0.8352
IVF+ICSI	62.84 (-192.63, 318.31)	0.6298	0.24 (-0.68, 1.16)	0.6092
Stage of embryos transferred				
D3	Ref		Ref	
D4	42.75 (-51.71, 137.21)	0.3752	-0.08 (-0.41, 0.25)	0.6303
D5	11.16 (-48.39, 70.71)	0.7134	0.07 (-0.14, 0.28)	0.4957
Number of embryos transferred				
1	Ref		Ref	
2	-13.14 (-69.28, 42.99)	0.6464	-0.06 (-0.25, 0.14)	0.5715
**Endometrial thickness (mm)**	16.09 (1.24, 30.93)	**0.0339**	0.06 (0.01, 0.11)	**0.0261**
Endometrial type				
A	Ref		Ref	
A-B	-1.04 (-247.40, 245.32)	0.9934	0.08 (-0.78, 0.95)	0.8501
B	-84.35 (-285.71, 117.01)	0.4118	-0.24 (-0.95, 0.46)	0.4980
B-C	-70.74 (-270.40, 128.91)	0.4875	-0.16 (-0.86, 0.53)	0.6467
C	-81.08 (-297.36, 135.21)	0.4626	-0.32 (-1.07, 0.44)	0.4099
Newborn sex				
Male	Ref		Ref	
Female	-26.60 (-82.85, 29.64)	0.3541	0.41 (0.21, 0.60)	**<0.0001**
**Gestational age (w)**	163.87 (150.99, 176.75)	**<0.0001**	--	--

COS, controlled ovarian stimulation; BMI, body mass index; AMH, antimüllerian hormone; E2, estradiol; LH, luteinizing hormone; CI, confidence interval; Ref, reference group. Bold values indicate: P *lt; 0.05, which is statistically significant.

The neonatal outcomes of the multivariate analyses are shown in **Table 3**. In the overall population, both PTD (adjusted OR: 1.89, 95% CI: 1.03 to 3.47; P = 0.0399) and LBW rates (adjusted OR: 2.67, 95% CI: 1.14 to 6.24; P = 0.0231) appeared to be positively associated with increasing progesterone level on hCG trigger day, even after accounting for confounding variables. Moreover, when progesterone level was taken as the categorical indicator, the odds of PTD (adjusted OR: 2.40, 95% CI: 1.11 to 5.19; P = 0.0267) and LBW (adjusted OR: 2.94, 95% CI: 1.06 to 8.14; P = 0.0383) still exhibited an increasing trend in the groups of progesterone level ≥1.0ng/ml, compared with the reference groups (progesterone <0.8ng/ml). However, when the patients were categorized into two groups according to the COS protocol, the results exhibited differences. In the GnRH antagonist protocol, the results were similar to those of the overall population. The incidence of both PTD (adjusted OR: 2.78, 95% CI: 1.08 to 8.12; P = 0.0268) and LBW (adjusted OR: 5.34, 95% CI: 1.16 to 9.55; P = 0.0314) significantly increased with rising progesterone levels on the hCG trigger day. The group with progesterone ≥ 1.0 ng/ml also exhibited significantly higher rates of PTD (adjusted OR: 4.54, 95% CI: 2.16 to 9.56; P = 0.0021) and LBW (adjusted OR: 4.16, 95% CI: 1.15 to 15.07; P = 0.0378) compared to the control group (progesterone <0.8ng/ml). However, these results were not observed in the GnRH agonist long protocol. Moreover, irrespective of whether the women were analyzed as a whole or stratified by the COS protocols, no statistically significant correlation was found between the progesterone level on the hCG trigger day and any of the following neonatal outcomes: birthweight, very LBW, fetal macrosomia, SGA, very SGA, LGA, very LGA, GA, or very PTD.

**Table 3:** Multivariable regression analysis for neonatal outcomes by progesterone level on hCG trigger day (n=2201).

Characteristics	Non-adjusted β/OR (95% CI)	*P* value	Adjusted β/OR (95% CI)	*P* value
TOTAL				
**Preterm (32-36w)**	1.76 (0.97, 3.18)	**0.0632**	1.89 (1.03, 3.47)	**0.0399**
Preterm (32-36w) (P level categorized into three groups)				
< 0.8 ng/ml	Ref		Ref	
0.8-1.0 ng/ml	1.37 (0.55, 2.28)	0.8372	1.23 (0.45, 2.03)	0.8456
≥ 1.0 ng/ml	2.35 (1.13, 4.90)	**0.0223**	2.40 (1.11, 5.19)	**0.0267**
**LBW (<2500g)**	2.82 (1.45, 5.48)	**0.0022**	2.67 (1.14, 6.24)	**0.0231**
LBW (<2500g) (P level categorized into three groups)				
< 0.8 ng/ml	Ref		Ref	
0.8-1.0 ng/ml	1.23 (0.54, 2.82)	0.6210	0.74 (0.24, 2.32)	0.6073
≥ 1.0 ng/ml	3.23 (1.31, 7.94)	**0.0107**	2.94 (1.06, 8.14)	**0.0383**
COS protocol= Antagonist protocol				
**Preterm (32-36w)**	2.33 (0.70, 7.83)	0.1700	2.78 (1.08, 8.12)	0.0268
Preterm (32-36w) (P level categorized into three groups)				
< 0.8 ng/ml	Ref		Ref	
0.8-1.0 ng/ml	1.27 (0.04, 2.43)	0.2030	1.24 (0.03, 2.28)	0.1765
≥ 1.0 ng/ml	6.56 (2.12, 9.08)	**0.0266**	4.54 (2.16, 9.56)	**0.0021**
**LBW (<2500g)**	4.88 (1.74, 8.66)	**0.0026**	5.34 (1.16, 9.55)	**0.0314**
LBW (<2500g) (P level categorized into three groups)				
< 0.8 ng/ml	Ref		Ref	
0.8-1.0 ng/ml	1.39 (0.29, 6.63)	0.6770	1.36 (0.02, 6.29)	0.4840
≥ 1.0 ng/ml	7.98 (1.72, 13.96)	**0.0079**	4.16 (1.15, 15.07)	**0.0378**
COS protocol= GnRH agonist long protocol				
**Preterm (32-36w)**	2.36 (0.94, 5.94)	0.0675	2.24 (0.82, 6.15)	0.1159
Preterm (32-36w) (P level categorized into three groups)				
< 0.8 ng/ml	Ref		Ref	
0.8-1.0 ng/ml	1.53 (0.74, 3.15)	0.2509	1.39 (0.62, 3.10)	0.4268
≥ 1.0 ng/ml	1.19 (0.49, 2.90)	0.6961	1.31 (0.53, 3.25)	0.5627
**LBW (<2500g)**	2.00 (0.64, 6.25)	0.2306	1.71 (0.35, 8.39)	0.5115
LBW (<2500g) (P level categorized into three groups)				
< 0.8 ng/ml	Ref		Ref	
0.8-1.0 ng/ml	1.18 (0.44, 3.13)	0.7455	0.95 (0.25, 3.63)	0.9412
≥ 1.0 ng/ml	1.93 (0.77, 4.84)	0.1604	2.18 (0.66, 7.15)	0.2004
TOTAL				
**Birthweight (g)**	-173.57 (-274.13, -73.02)	**0.0007**	-80.54 (-169.69, 8.61)	0.0769
**Z-score**	-0.27 (-0.49, -0.06)	**0.0117**	-0.13 (-0.35, 0.10)	0.2748
**Very LBW (<1500g)**	3.18 (0.45, 22.44)	0.2453	5.93 (0.59, 60.00)	0.1316
**Fetal macrosomia (≥4000g)**	0.37 (0.16, 0.84)	**0.0181**	0.49 (0.20, 1.19)	0.1132
**LGA (>90^th^ percentile)**	0.39 (0.17, 0.88)	**0.0241**	0.43 (0.17, 1.06)	0.0653
**Very LGA (>97^th^ percentile)**	0.29 (0.09, 0.93)	**0.0368**	0.48 (0.14, 1.67)	0.2490
**SGA (<10^th^ percentile)**	1.45 (0.62, 3.42)	0.3914	1.01 (0.38, 2.68)	0.9784
**Very SGA (<3^rd^ percentile)**	0.65 (0.13, 3.26)	0.5997	0.69 (0.10, 4.58)	0.7016
**Gestational age (w)**	-0.33 (-0.68, 0.03)	0.0715	-0.32 (-0.68, 0.04)	0.0810
**Very preterm (<32w)**	1.16 (0.15, 8.72)	0.8872	1.97 (0.22, 17.73)	0.5469
**Newborn height**	-0.48 (-0.97, -0.00)	**0.0499**	-0.38 (-0.91, 0.15)	0.1553
**Newborn sex**	0.91 (0.62, 1.35)	0.6450	0.89 (0.58, 1.38)	0.6127
**Mode of delivery**	1.22 (0.81, 1.85)	0.3405	1.24 (0.78, 1.96)	0.3659

Analyses of birthweight, fetal macrosomia, LBW, very LBW, SGA, very SGA, LGA and very LGA were adjusted for maternal BMI, AMH, infertility duration, infertility cause, COS protocols, dosage of Gn, E2 level on hCG day, endometrial thickness, stage of embryos transferred, gestational age and newborn sex.

Analyses of GA, preterm and very preterm were adjusted for maternal BMI, AMH, infertility duration, infertility cause, COS protocols, endometrial thickness, stage of embryos transferred and newborn sex.

COS, controlled ovarian stimulation; GA, gestational age; PTD, preterm delivery; LBW, low birth weight; SGA, small for gestational age; LGA, large for gestational age; OR, odds ratio; CI, confidence interval; Ref, reference group. Bold values indicate: P < 0.05, which is statistically significant.

The curves in **Figures 2** and **3** showed the relationship between the progesterone level on the hCG trigger day and the adjusted incidence of PTD and LBW in different COS protocols, respectively. Combined with the threshold effect analysis using piece-wise linear regression as displayed in **Table 4**, the results indicated that in the GnRH antagonist protocol, when the progesterone level was > 0.90 ng/ml, PTD rates increased significantly with increasing progesterone levels (adjusted OR: 6.60, 95% CI: 2.52 to 14.56; P = 0.0131). Additionally, when the progesterone level was > 0.98 ng/ml, the incidence of LBW increased noticeably with increasing progesterone levels (adjusted OR: 7.50, 95% CI: 3.71 to 15.87; P = 0.0038). However, in the GnRH agonist long protocol, no significant differences in PTD or LBW were observed across progesterone level groups. The threshold effect analysis identified 0.90 ng/mL as the inflection point at which progesterone levels began to affect neonatal outcomes, and this effect was only observed in the GnRH antagonist protocol.

**Figure 2:**
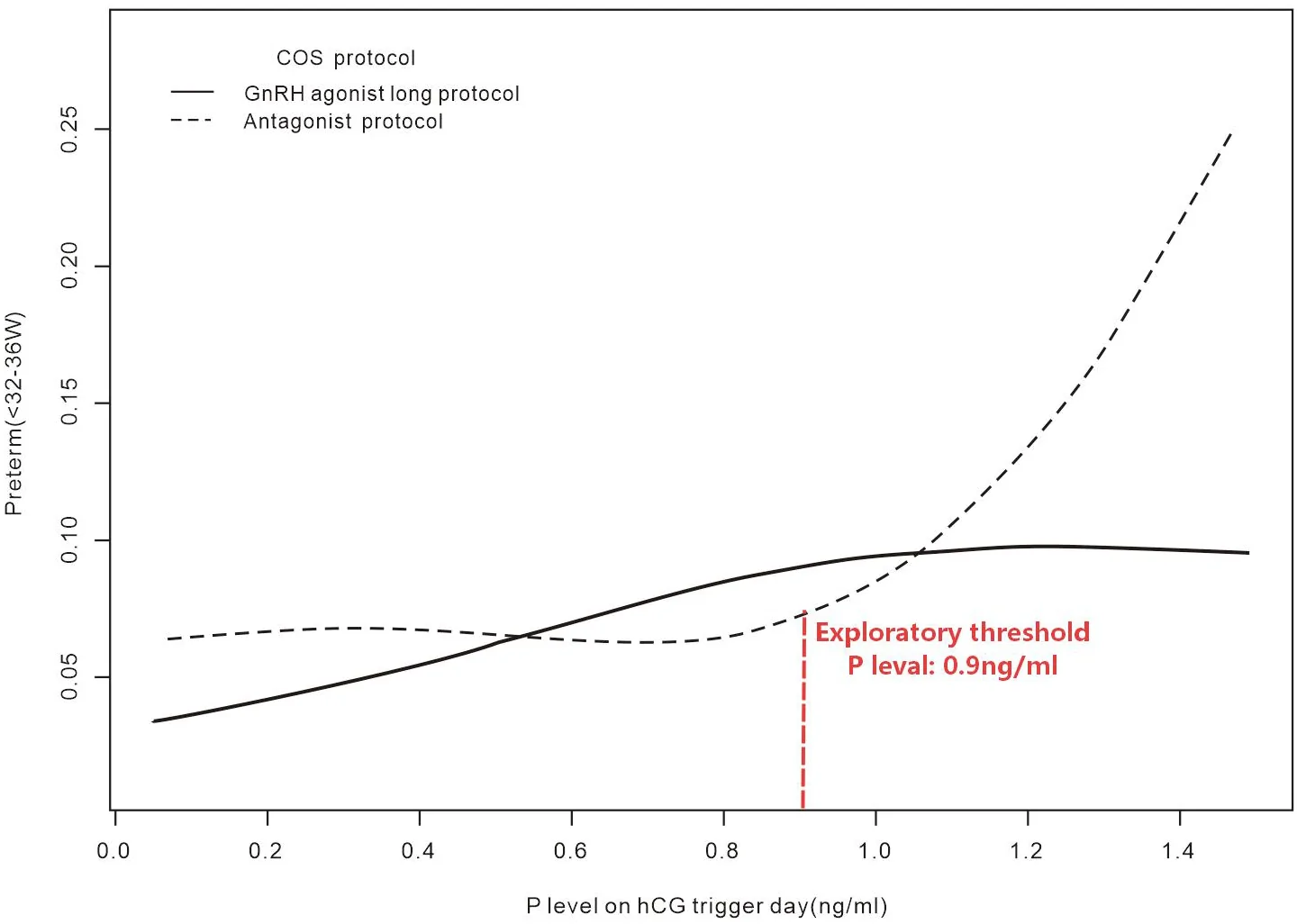
The relationship between the progesterone level on hCG trigger day and the adjusted incidence of preterm delivery across different COS protocols. Analyses were adjusted for maternal BMI, AMH, infertility duration, infertility cause, COS protocols, endometrial thickness, stage embryo transferred and gender of newborn. P, progesterone; COS, controlled ovarian stimulation. P, progesterone; COS, controlled ovarian stimulation.

**Figure 3:**
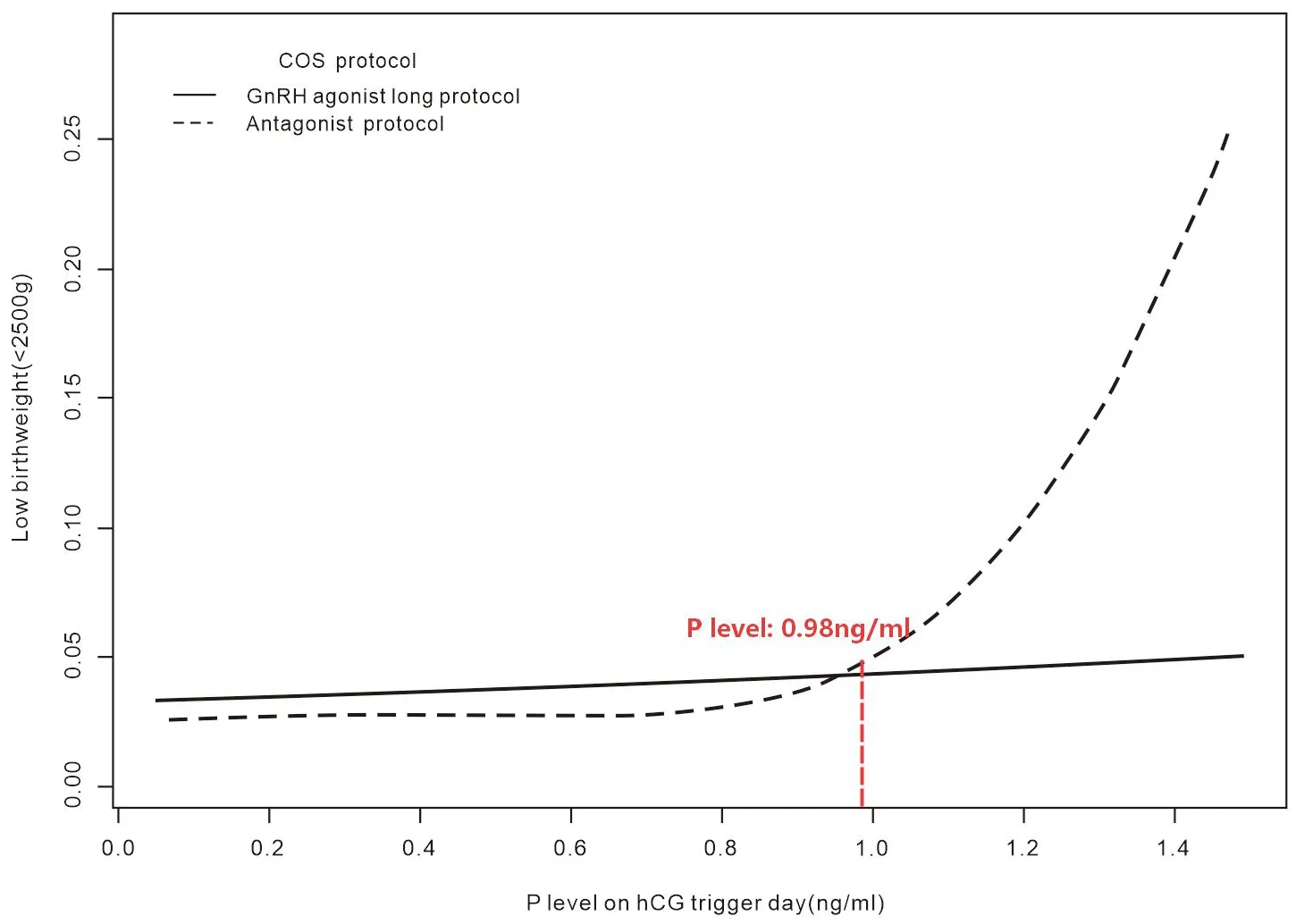
The relationship between the progesterone level on hCG trigger day and the adjusted incidence of low birthweight across different COS protocols. Analyses were adjusted for maternal BMI, AMH, infertility duration, infertility cause, COS protocols, dosage of Gn, E2 level on HCG day, endometrial thickness, stage embryo transferred, gestational age and gender of newborn. P, progesterone; COS, controlled ovarian stimulation.

**Table 4:** Threshold effects of progesterone level on the incidence of preterm and LBW using piece-wise logistic regression. .

Characteristics	Non-adjusted OR (95% CI)	*P* value	Adjusted OR (95% CI)	*P* value
Preterm (32-36W)				
Antagonist protocol				
Progesterone level ≤ 0.90 ng/ml	0.49 (0.07, 3.23)	0.4552	0.76 (0.11, 5.27)	0.7820
Progesterone level > 0.90 ng/ml	7.74 (2.47, 12.53)	**0.0116**	6.60 (2.52, 14.56)	**0.0131**
GnRH-a long protocol				
Progesterone level ≤ 0.90 ng/ml	1.97 (0.54, 7.12)	0.3026	2.22 (0.41, 7.26)	0.4631
Progesterone level > 0.90 ng/ml	1.81 (0.11, 8.01)	0.6808	1.59 (0.17, 8.68)	0.3846
LBW (<2500g)				
Antagonist protocol				
Progesterone level ≤ 0.98 ng/ml	1.27 (0.01, 7.25)	0.4380	1.04 (0.02, 12.95)	0.6367
Progesterone level> 0.98 ng/ml	8.08 (3.22, 13.24)	**0.0016**	7.50 (3.71, 15.87)	**0.0038**
GnRH-a long protocol				
Progesterone level ≤ 0.98 ng/ml	1.88 (0.33, 10.82)	0.4793	1.03 (0.10, 10.71)	0.9816
Progesterone level> 0.98 ng/ml	0.36 (0.01, 13.43)	0.7386	1.12 (0.03, 14.29)	0.4285

Analyses of preterm (32-36W) were adjusted for maternal BMI, AMH, infertility duration, infertility cause, COS protocols, endometrial thickness, stage of embryos transferred and newborn sex.

Analyses of LBW were adjusted for maternal BMI, AMH, infertility duration, infertility cause, COS protocols, dosage of Gn, E2 level on HCG day, endometrial thickness, stage of embryos transferred, gestational age and newborn sex.

GnRH-a, GnRH agonist; LBW, low birthweight; OR, odds ratio; CI, confidence interval. Bold values indicate: P < 0.05, which is statistically significant.

## Discussion

The main objective of our current study, which included 2, 201 singleton live births from infertile women aged under 42 years, was to evaluate the relationship between serum progesterone levels on the day of hCG trigger and neonatal outcomes following fET. After adjusting for potential confounding factors, we observed a strong positive correlation between progesterone levels and the incidence of PTD and LBW in the GnRH antagonist protocol, regardless of whether progesterone level was treated as a continuous or categorical variable. Furthermore, the results of adjusted curve fitting and threshold effect analysis showed that the exploratory threshold value of progesterone level was 0.90 ng/mL. This finding suggests that in the GnRH antagonist protocol, a progesterone level exceeding 0.90 ng/mL may be an independent predictor of an increased risk of PTD in singletons born after fET. In contrast, in the GnRH agonist long protocol, no significant association was found between progesterone levels on the day of hCG administration and neonatal outcomes within the range of progesterone level < 1.5 ng/mL (cycles with progesterone > 1.5 ng/mL were canceled for fET in our center). This may indicate that, in the antagonist protocol, a lower progesterone threshold leads to an increased risk of adverse neonatal outcomes, whereas in the GnRH-a long protocol, this threshold may be higher. To the best of our knowledge, this is the first study to establish an exploratory threshold for the impact of progesterone levels on neonatal outcomes through smooth curve fitting and threshold effect analysis using piece-wise linear regression, with a stratified comparative analysis between different COS protocols.

In recent years, there has been a growing concern about the safety of maternal and newborns following IVF treatment, which extends beyond pregnancy outcomes alone. However, the majority of research on progesterone levels on the hCG trigger day during COS has primarily focused on live birth rate, with very limited study exploring their correlation with neonatal outcomes. Given the scarcity of research in this area, both Ibrahim’s study and ours indicated that elevated progesterone levels had a negative impact on neonatal outcomes. The crucial distinction lies in the findings of Ibrahim et al., which showed a significant reduction in birthweight when progesterone levels exceeded 2.0 ng/mL (22). In contrast, our study revealed a significant increase in the incidence of PTD when progesterone levels surpassed 0.9 ng/mL in the GnRH antagonist protocol. These two different thresholds may mainly stem from the fact that Ibrahim’s study used empirical grouping, while our study employed smooth curve fitting and threshold effect analysis, along with stratification by COS protocols. Additionally, our data also initially showed a significant decrease in birthweight. However, after adjusting for confounding factors in multivariate regression analysis, we found no statistically significant differences in birthweight among the different progesterone level groups. These differences may be attributed to the different confounding factors adjusted for in each study. The progesterone models in the study by Ibrahim et al. were adjusted for maternal age and peak estradiol levels (22). Our analysis in addition to these two factors, also adjusted for maternal BMI, AMH, duration of infertility, cause of infertility, COS protocols, Gn dosage, endometrial thickness, stage of embryos transferred, GA, and neonatal sex, all of which may potentially interfere with the results. Subsequently, a study involving 499 newborns found that elevated serum progesterone level within the range of 0.64–1.05 ng/mL on the day of hCG administration was associated with a mildly increased risk of ischemic placental disease following fET. This may be one of the contributing factors to the increased incidence of PTD and LBW (26).

Previous studies on progesterone thresholds for pregnancy outcomes have reported varying cutoff values, with some setting the threshold at 1.0 ng/mL (2, 8, 27), others at 1.5 ng/mL (28–30), and still others at 2.0 ng/mL (22, 31). A large meta-analysis of over 60, 000 cycles indicated that higher progesterone levels using a threshold of ≥ 0.8 ng/mL were significantly associated with a decreased probability of pregnancy in fresh IVF cycles (9). Seon et al’ s data showed that a serum progesterone concentration of 0.9 ng/mL on the day of hCG trigger may serve as a threshold level that significantly negatively impacts IVF pregnancy outcomes in GnRH antagonist protocols (32), which is consistent with our findings. Notably, all of the aforementioned studies focused on the association between progesterone levels and pregnancy outcomes, such as live birth rate and clinical pregnancy rate, whereas exploratory studies on the threshold of progesterone levels in relation to neonatal outcomes, including GA, PTD, birthweight, and LBW, are essentially lacking. Moreover, most of the progesterone thresholds in the aforementioned studies were determined by empirical grouping at their respective centers, whereas our study employed smooth curve fitting and threshold effect analysis to calculate the cutoff value, aiming to provide clinicians with some hints and guidance in assessing the impact of progesterone levels on neonatal birth outcomes.

Our center’s current policy is to cancel fET when progesterone on the hCG trigger day exceeds 1.5 ng/mL; therefore, our conclusions are strictly limited to patients with P ≤ 1.5 ng/mL who underwent fET. Notably, this study found that in GnRH antagonist cycles, even when P is below 1.5 ng/mL, the risk of preterm birth is significantly increased once P exceeds 0.90 ng/mL. This suggests that in antagonist cycles, even when P is between 0.90 and 1.5 ng/mL and transfer is not canceled, clinicians should remain vigilant about neonatal risks and strengthen prenatal monitoring. However, whether the current 1.5 ng/mL cancellation threshold should be re-evaluated can only be determined after prospective multicenter studies including a broader range of progesterone levels are conducted. Until higher-level evidence is available, the thresholds proposed in this study should be regarded as exploratory and should not directly change clinical practice.

Investigators have linked the progesterone level on the hCG trigger day to multiple follicular development and increased ovarian steroidogenic activity (33). Furthermore, it is believed that the progesterone level at the end of COS depends on the ovarian response, tending to be higher in patients with a high ovarian response (34). In this study, patients were of similar age and had comparable LH levels on the hCG administration day. The group with a higher progesterone level had higher number of oocytes and higher peak E2 levels, consistent with previous literature reports (33, 34). Considering the impact of E2 levels on birthweight (35), we adjusted for E2 level in multiple regression analysis to minimize its interference with neonatal outcomes. In this study, a cutoff progesterone level of 0.9 ng/ml was identified, which significantly increased the incidence of PTD in GnRH antagonist cycles, while no such findings was observed in GnRH agonist protocols. Why was the outcome like this? Previous studies have found that, compared with patients receiving GnRH agonist protocol and natural cycle protocol, patients receiving GnRH antagonist protocol are more likely to exhibit abnormal expression of multiple endometrial receptivity molecular markers (36), inhibited expression of the c-kit receptor in endometrial stromal cells (36), and significantly decreased expression level of S100P protein in endometrial epithelial cells (13), ultimately leading to a greater susceptibility to decreased endometrial receptivity and displacement of the window of implantation. Therefore, at the same progesterone level on hCG trigger day, the antagonist protocol may pose a greater threat to endometrial receptivity compared to the agonist protocol, which may explain why the antagonist protocol is more prone to induce PTD and LBW births.

The exact mechanism underlying the increased incidence of PTD and LBW in pregnancies resulting from fET has not been clearly elucidated. Given that the phenotypes observed in this study—PTD and LBW—are closely related to placental/uterine function, the leading hypothesis is that premature progesterone elevation induces premature endometrial transformation, prematurely closing the receptivity window and potentially affecting early placentation and spiral artery remodeling, thereby increasing placenta-related adverse outcomes. Endometrial biopsies and ultrasound assessment of the endometrium have confirmed that elevated progesterone level causes premature secretory transformation of the endometrium, leading to an asynchrony at the embryo-endometrium crosstalk, which in turn impairs the implantation process (8, 11, 12). Endometrial biopsies have also revealed altered regulation for 140 genes in women with elevated progesterone level during follicular phase leading to changes in gene expression (37, 38). Another explanation for the observed phenomena would be impaired oocyte/embryo quality, as elevated progesterone may reflect an abnormal intrafollicular environment affecting oocyte maturation and developmental potential; impaired trophoblast development could then indirectly lead to placental dysfunction and adverse neonatal outcomes. Some studies have reported that premature progesterone elevation yields fewer embryos beyond the four-cell stage, fewer good-quality embryos, and lower implantation rates (21), and that progesterone levels >2.0 ng/mL during the follicular phase adversely affects oocyte and top-quality embryo rates (18, 20); a retrospective analysis of over 3, 400 antagonist ICSI cycles also showed increased embryo wastage and reduced cumulative live birth rates (19). In contrast, evidence from frozen-thawed embryo transfer suggests that embryos from cycles with elevated progesterone on the hCG day perform better when transferred in frozen rather than fresh cycles (39, 40), and meta-analyses indicate that the adverse impact of elevated progesterone is mainly due to altered endometrial regulation rather than oocyte or embryo quality (9). Thus, the evidence on embryo quality remains inconsistent. Because this study is retrospective and did not perform molecular analyses of embryo or placental tissue, we cannot distinguish the relative contributions of endometrial versus embryonic/placental mechanisms. Further studies combining embryo morphology, trophoblast markers, and placental pathology are warranted to clarify the mechanisms linking elevated progesterone to adverse birth outcomes.

This is the first study to establish an exploratory threshold for the impact of progesterone levels on neonatal outcomes through smooth curve fitting and threshold effect analysis using piece-wise linear regression, with stratification by COS protocols. Its main strength lies in reporting an approximate threshold of 0.90 ng/mL for progesterone levels on the day of hCG administration, above which the incidence of PTD is significantly increased. This provides new evidence to the limited body of research on the relationship between progesterone levels on the day of hCG trigger and neonatal outcomes following fET. However, the power of this study is limited by its retrospective design, which may introduce selection bias and residual confounding. Second, differences in progesterone assay methods may affect the generalizability of the thresholds; samples in this study were measured at the same center using the same platform, but the approximately 0.90 ng/mL threshold in antagonist cycles may not be directly extrapolated to centers using other assay platforms. Third, The policy at our center is to cancel fET when progesterone levels exceed 1.5 ng/mL. Therefore, the current results are only applicable to patients with progesterone ≤ 1.5 ng/mL; further studies are needed for those with progesterone > 1.5 ng/mL. Moreover, PCOS patients were excluded to minimize confounding from PCOS-related metabolic and endocrine abnormalities, which limits the applicability of our findings to this population, as PCOS patients often have higher ovarian responses and different progesterone profiles. Therefore, the thresholds proposed in this study should be regarded as exploratory and require prospective multicenter validation before clinical implementation.

## Conclusion

In conclusion, in singletons born to patients under 42 years old following fET, there is a strong positive correlation between progesterone levels on the day of hCG administration and the incidence of PTD and LBW. Our data further demonstrated that a progesterone level > 0.90 ng/ml may be an independent predictor for greater odds of PTD after fET in the GnRH antagonist protocol, whereas this correlation was not observed in the long agonist protocol. Therefore, we suggest that women with higher progesterone levels after conception via fresh antagonist cycles should receive more attention from obstetricians and pediatricians to reduce the incidence of PTD and LBW. However, given the variability in progesterone level measurements across different centers, large-sample, multicenter, prospective studies are needed in the future to further investigate this issue.

## Data Availability

The raw data supporting the conclusions of this article will be made available by the authors, without undue reservation.
